# Overexpression of TC2N is associated with poor prognosis in gastric cancer

**DOI:** 10.7150/jca.50653

**Published:** 2021-01-01

**Authors:** Jianbo Xu, Xinde Ou, Jin li, Qinbo Cai, Kaiyu Sun, Jingning Ye, Jianjun Peng

**Affiliations:** 1 Department of Gastrointestinal Surgery, the First Affiliated Hospital, Sun Yat-sen University, 58 Zhongshan 2nd Road, Guangzhou 510080, China.; 2 Digestive Disease Center, the Seventh Affiliated Hospital, Sun Yat-sen University, 628 Zhenyuan Road, Shenzhen 518000, China.; 3 Laboratory of General Surgery, the First Affiliated Hospital, Sun Yat-sen University, 58 Zhongshan 2nd Road, Guangzhou 510080, China.

**Keywords:** TC2N, Gastric cancer, Prognosis

## Abstract

**Background:** Tac2-N (TC2N) is a tandem C2 domain-containing protein, acting as a novel oncogene or suppressor in different kinds of cancers. However, the status of *TC2N* expression and its significance in gastric cancer (GC) is still unclear. The present study is aimed to elucidate the clinicopathological significance and prognostic value of *TC2N* level in GC.

**Methods:** We used sequencing data from the Cancer Genome Atlas (TCGA) database to analyze *TC2N* expression in GC by UALCAN database and Gene Expression Profiling Interactive Analysis tools (GEPIA). *TC2N* expression level in 12 pairs of fresh GC tissues and adjacent nontumorous tissues was detected by quantitative real-time reverse-transcription polymerase chain reaction (RT-PCR) and Western blot (WB) assays. Immunohistochemical (IHC) staining was used to detect TC2N protein expression in Paraffin-embedded tissues in our center. *In vitro* proliferation, migration and invasion assays were used to evaluate the effect of *TC2N* on functional capability of gastric cancer cells. LinkedOmics was used to identify gene expressions associated with *TC2N*.

**Results:** The mRNA and protein expression of *TC2N* in gastric cancer were both significantly higher than normal gastric mucosa. It was also elevated in gastric cancer cells compared with normal gastric epithelium cell. *In vitro* assays suggested that *TC2N* facilitated proliferation, migration and invasion of gastric cancer cells. Bioinformatic analysis showed a widespread impact of *TC2N* on the transcriptome and a strong interaction with tumor associated genes. We also found that *TC2N* was an independent prognostic factor for long-term survival in GC patients and its high expression was evidently associated with poor overall survival and recurrence-free survival.

**Conclusions:** Our results show that high level of TC2N correlates with poor prognosis in patients with gastric cancer and promotes the development of gastric cancer. Thus, TC2N expression can serve as a prognostic biomarker for patients with gastric cancer.

## Introduction

As the fifth most common cancer worldwide, gastric cancer (GC) is still incurable and induces 782,685 related death occurs in 2018 1, 2. A large quantity of evidence has shown that GC remains an excessive health burden in several Asian countries, especially in China, Korea and Japan 3, 4. Despite the development of surgical techniques and the improvement of chemotherapy and radiotherapy regimens, gastric cancer, especially advanced gastric cancer, is still a dreadful disease with high mortality 5-8. Due to the fact that convenient and effective methods in predicting the prognosis of patients with gastric cancer are still lacking 9, 10, novel biomarkers for improving early diagnosis, prognostic evaluation and tumor grading of GC are urgently needed.

*Tac2-N* (*TC2N*), located on human chromosome 14q32.12, encodes a putative C2 domain-containing protein that belongs to the carboxyl-terminal type (C-type) tandem C2 protein family. TC2N was first cloned in the mouse and contains two C-terminal C2 domains, C2A and C2B 11. The C2 domain was originally identified as a protein structural domain of calcium-dependent protein kinase C 12-14. Further studies indicated that the function of the C2 domain is not limited to calcium-dependent phospholipid binding since this motif has been implicated in cellular signal transduction and protein-protein interactions 15. A number of proteins containing C2 domains are involved in the regulation of tumorigenesis. It is indicated that *TC2N* accelerates tumor progression in lung cancer by different mechanisms 16, 17. Moreover, *TC2N* can also inhibit cell proliferation and tumor growth and correlates with a better prognosis in breast cancer 18. However, the role *TC2N* playing in gastric cancer is still unclear.

The present study was undertaken to assess *TC2N* expression in GC specimens and explore its role in GC, and therefore to evaluate whether TC2N can serve as a prognostic biomarker for patients with GC.

## Materials and Methods

### Patients and specimens

Fresh gastric cancer and paired adjacent normal gastric mucosa tissues from resection specimens were collected from patients with primary gastric cancer who were treated by total or partial gastrectomy at the First Affiliated Hospital of Sun Yat-sen University (FAHSYSU) in 2017 (N = 12). All separated tissues were frozen immediately in liquid nitrogen and then stored in liquid nitrogen. Paraffin-embedded tissues of gastric cancer and normal gastric mucosa were obtained from the Department of Pathology at the FAHSYSU. A total of 232 gastric cancer patients who underwent gastrectomy in our center were enrolled in this study. None of the patients received chemotherapy or other treatments before sampling. The pathological diagnoses and classifications were made according to the American Joint Committee on Cancer (AJCC) cancer staging manual, 7^th^ ed 19. Ethical approval for clinical research was obtained from the Institutional Review Board of FAHSYSU.

### Cell culture

Gastric cancer cell lines, including AGS, MKN28 and HGC27, were obtained from the Type Culture Collection of Chinese Academy of Sciences (Shanghai, China). The normal gastric epithelial cell line GES1 was purchased from the Lab Animal Center of the Fourth Military Medical University (Shanxi, China). Cells were maintained in DMEM (Invitrogen, USA) supplemented with 10% fetal bovine serum (GBICO, USA) in incubator with 5% CO2 and 95% room air at 37°C.

### RNA isolation and reverse transcription-quantitative real-time PCR (RT-qPCR)

RNA was isolated from the cell lines using the RNA isolation plus (TaKaRa, Japan). Single-stranded cDNA was generated from 1μg total RNA in a 20μL reaction volume using oligodT primers according to the protocol supplied with the Primer Script TM RT Reagent (TaKaRa, Japan). The relative expression levels were measured by qPCR using the ABI 7900HT instrument (Applied Biosystems, USA) in a total volume of 10ul with the SYBR green detection system (Takara, Japan) and GAPDH was used as an endogenous control. Oligonucleotide sequences of the primer sets used were as follows: *TC2N* (forward: 5′ TGGCTGTACTGAGGATTATTTGC-3′, reverse: 5′- TGTGAAGGAGTTTCTTGTGTCC-3′); *GAPDH* (forward: 5′-ACAACTTTGGTATCGTGGAAGG-3′, reverse: 5′-GCCATCACGCCACAGTTTC-3′).

### Western blot

For western blot analysis, total proteins from cell lines and fresh-frozen tissues were extracted using the Whole Cell Protein Extraction Kit (KeyGEN, China). BCA Protein Quantitation Assay (KeyGEN, China) was used to determine the protein concentration. An equal amount (30 μg) of protein sample was separated on 10% sodium dodecyl sulfate-polyacrylamide gels (SDS-PAGE) and transferred to polyvinylidene fluoride membranes (PVDF) (Millipore, USA). The membranes were then blocked with 5% non-fat dry milk in Tris-buffered saline (TBS)/0.1% Tween 20 for 1 h at room temperature. Membranes were incubated with anti-TC2N (1:1000, PA5-26433, Invitrogen, USA), anti-β-actin (1:1000, 14395-1-AP, Proteintech, China,) primary antibodies overnight at 4 °C. The next day membranes were washed and incubated with horseradish peroxidase-conjugated secondary antibody. Proteins were visualized using Immobilon Western Chemiluminescent HRP Substrate (Millipore, USA).

### Immunohistochemical staining and evaluation

The paraffin-embedded tissues were subjected to standard Immunohistochemical (IHC) staining, followed by streptavidin-biotin-peroxidase complex method as previous described 20. In brief, slides were deparaffinized and then heated for antigen retrieval in EDTA buffer (pH 8.9) at 105 °C for 5 min and natural cooled for 30 min. Endogenous peroxidase activity was blocked in 3% hydrogen peroxide for 10 min. After blocked in 10% normal goat serum (KeyGen, China) for 30 min, sections were incubated with rabbit polyclonal anti-TC2N antibody (1:100, PA5-26433, Invitrogen, USA) at 4° overnight. EnVision Plus System-HRP (DAKO, Denmark) was used according to manufacturer's instruction and targeted protein was visualized using diaminobenzidine (DAB) as the substrate. And the nucleus was displayed by Mayer's hematoxylin counterstaining 21.

IHC results evaluation was performed by two double-blinded independent observers who were unaware of the clinical and pathological characteristics associated with the specimens, and scoring was determined using a semiquantitative method as previously described 22. Samples in which more than 10% of the tumor cells were stained were considered positive. The staining intensity was defined as follows: 0 (negative), 1 (weak), 2 (moderate), and 3 (strong). Negative and weak staining was considered to indicate low TC2N expression, and moderate and strong staining was considered to indicate high TC2N expression.

### RNA interference and Plasmid construction

Two different sequences of *TC2N* siRNA, including negative control siRNA, were designed and chemically synthesized by Ribobio (China). These two siRNA candidates were listed as follows: si-001 (targeting GGATAAGTGAAGACAGTAA) and si-002 (targeting GCACGGACCTAGCTATGAT). The expression vector encoding full-length open reading frame of human *TC2N* were constructed by synthesis and inserted into p3xFLAG-CMV-14. SiRNAs or plasmid were transfected to cells with the Lipofectamine 3000 reagent (Invitrogen, USA) following the manufacturer protocol.

### Cell Counting Kit-8 assay

The Cell Counting Kit-8 (CCK8, Dojindo, Japan) was used according to the manufacturer's instructions. Briefly, 1,000 cells/wells were seeded in a 96-well plate and 10 μl of CCK8 reagent were mixed with 90 μl of fresh medium to replace the original medium. Wells were further incubated for 3 h before being tested.

### Cell migration and invasion assays

GC Cell migration and invasion were evaluated by conducting the Transwell assay, according to a previously described method 22. Transwell inserts (pore size, 8 μm; BD Biosciences, USA) were specially precoated with Matrigel (BD Biosciences, USA) for use in the invasion assays. Infected AGS and MKN28 cells were harvested and 1 × 10^5^ cells were suspended in 200 μL of serum-free medium and placed in a Transwell insert. The lower chamber was filled with 400 μL of medium containing 20% FBS. After the cells were incubated for 24 h at 37 °C, cells on the underside of the membrane were fixed with 4% paraformaldehyde for 15 min and subsequently stained with 0.1% crystal violet in 20% ethanol. Cell counts were performed for five randomly selected fields at ×100 magnification using phase contrast microscopy (Olympus, Germany). Transwell assays were replicated three times.

### Database analysis

The mRNA expressions and tumor subgroup gene expressions of *TC2N* in GC tissues and normal gastric tissues were analyzed using the sequencing data from the Cancer Genome Atlas (TCGA) database 23 by UALCAN database 24 and Gene Expression Profiling Interactive Analysis tools (GEPIA) 25. The LinkedOmics database 26 is a Web-based platform for analyzing 32 TCGA cancer-associated multi-dimensional datasets. The LinkFinder module of LinkedOmics was used to study genes differentially expressed in correlation with *TC2N* in the TCGA. Results were analyzed statistically using Spearman or Pearson's correlation coefficient. Protein-protein interaction (PPI) networks were predicted using the Gene MANIA 27 and STRING program 28.

### Statistical analysis

Statistical analysis and graphic plotting were performed using SPSS Statistics version 20.0 (IBM, USA) and GraphPad Prism version 6.0 (GraphPad Software, USA). Difference of *TC2N* mRNA expressions between groups was analyzed using Student's t-test. The significance of associations between TC2N expression and clinicopathological features was analyzed using the Chi-square test. Survival curves were plotted using Kaplan-Meier method and the differences between groups were compared using log-rank test. Hazards ratio for clinicopathological characteristics in univariate and multivariate analysis were both estimated with Cox proportional hazards regression. Two tailed test was used for all the statistical analysis and *P*-value < 0.05 was considered statistically significant.

## Results

### The expression of *TC2N* is elevated in gastric cancer

**Figure 1:**
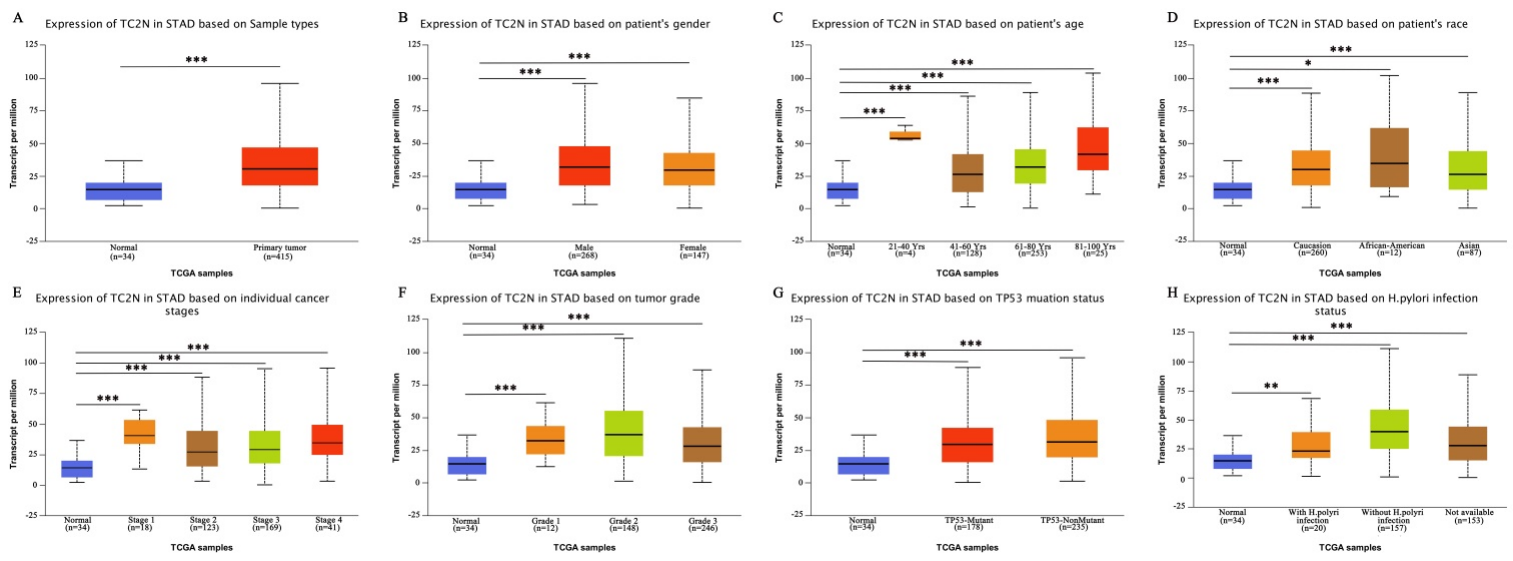
** TC2N transcription in subgroups of patients with gastric cancer (UALCAN).** (A) Boxplot showing relative expression of TC2N in normal and GC samples. (B) Boxplot showing relative expression of TC2N in normal individuals of either gender or male or female GC patients. (C) Boxplot showing relative expression of TC2N in normal individuals of any age or in GC patients aged 21-40, 41-60, 61-80, or 81-100 yrs. (D) Boxplot showing relative expression of TC2N in normal individuals of any ethnicity or in GC patients of Caucasian, African-American or Asian ethnicity. (E) Boxplot showing relative expression of TC2N in normal individuals or in GC patients in stages 1, 2, 3 or 4. (F) Boxplot showing relative expression of TC2N in normal individuals or GC patients with grade 1, 2 or 3 tumors. (G) Boxplot showing relative expression of TC2N in normal individuals or GC patients with or without TP53 mutation. (H) Boxplot showing relative expression of TC2N in normal individuals or GC patients with or without H.pylori infection. Data are mean ± SE. *, *P* < 0.05; **, *P* < 0.01; ***, *P* < 0.001.

**Figure 2:**
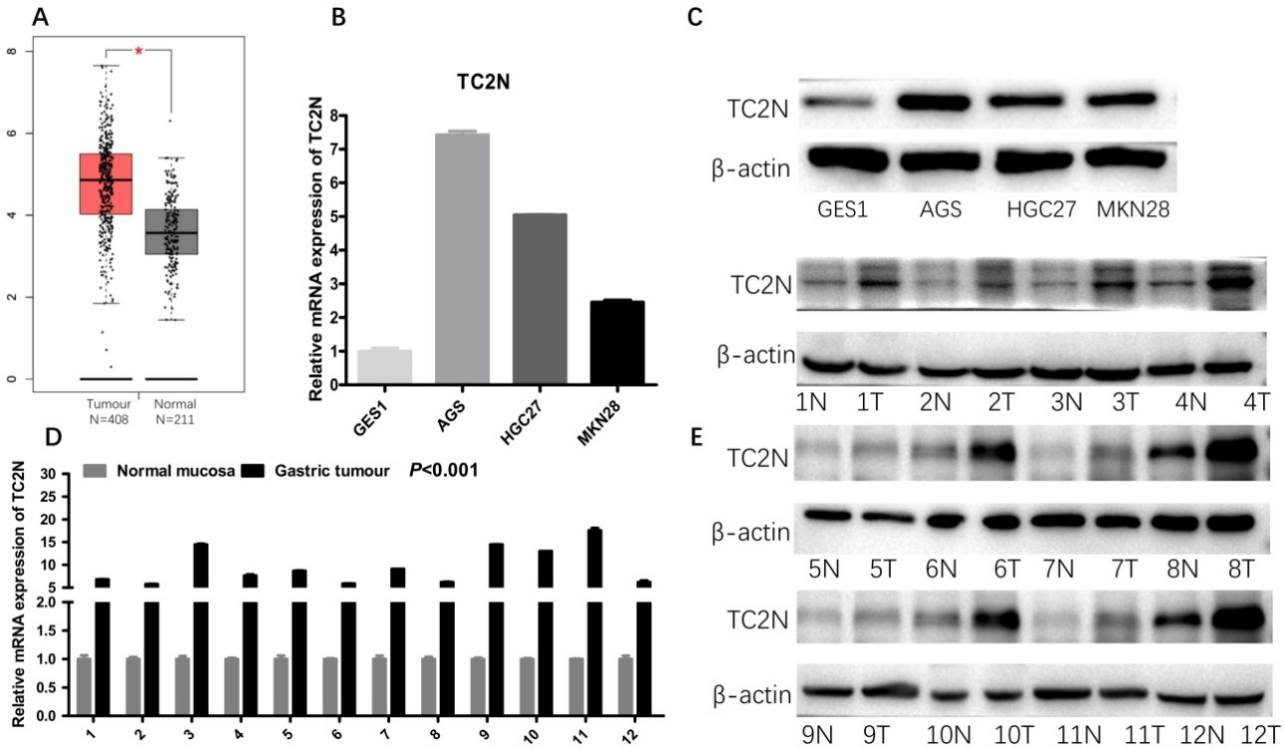
** High *TC2N* expression in gastric cancer.** (A) Analysis of *TC2N* expression in unpaired GC (T = 408) and normal tissues (N = 211) in the GEPIA (*P*<0.05). (B, C) The measurements of *TC2N* expression in gastric cancer cell lines compared with the normal cell line GES1 by qRT-PCR(B) and WB(C). (D, E) The measurements of *TC2N* expression in 12 paired GC tissues and matched normal adjacent mucosa, analyzed by qRT-PCR(D) and WB(E). T, GC tissues; N, matched normal adjacent mucosa. *, *P* < 0.05.

Firstly, we analyzed *TC2N* transcription levels in multiple GC studies from TCGA. Data in the UALCAN database revealed that the mRNA expression of *TC2N* was significantly higher in GC tissues than in normal tissues (*P <* 0.001) (Figure 1A). Further sub-group analysis of multiple clinic pathological features of GC samples in the TCGA consistently showed that the transcription levels of *TC2N* were significantly higher in GC tissue than normal tissue in subgroup analyses based on gender, age, ethnicity, disease stages, tumor grade, *TP53* mutation and H.pylori infection (Figure 1B-H). The mRNA level of *TC2N* in GC tissues and normal gastric tissues from the GEPIA database also showed an increase in gastric cancers compared with normal gastric tissues (Figure 2A). Next, RT-qPCR was conducted on RNA samples derived from several gastric cancer cell lines and normal gastric epithelium cell line GES1. When compared with GES1, it was found that *TC2N* mRNA expressions were higher in AGS, MKN28 and HGC27 (Figure 2B) and the tendency of protein level tested by western blot was in accordance with the RNA level (Figure 2C). To further confirm the *TC2N* expression in gastric cancer samples, *TC2N* was detected by RT-qPCR and WB in 12 pairs of gastric cancer tissues and adjacent normal gastric mucosa. We found that both the mRNA and protein levels of *TC2N* were markedly elevated in gastric cancer tissues in comparison with adjacent normal gastric tissues (Figure 2D, E). Thus, TC2N expression may serve as a potential diagnostic indicator in GC.

### Relationships between the expression of TC2N and clinicopathological features in gastric cancer

**Figure 3:**
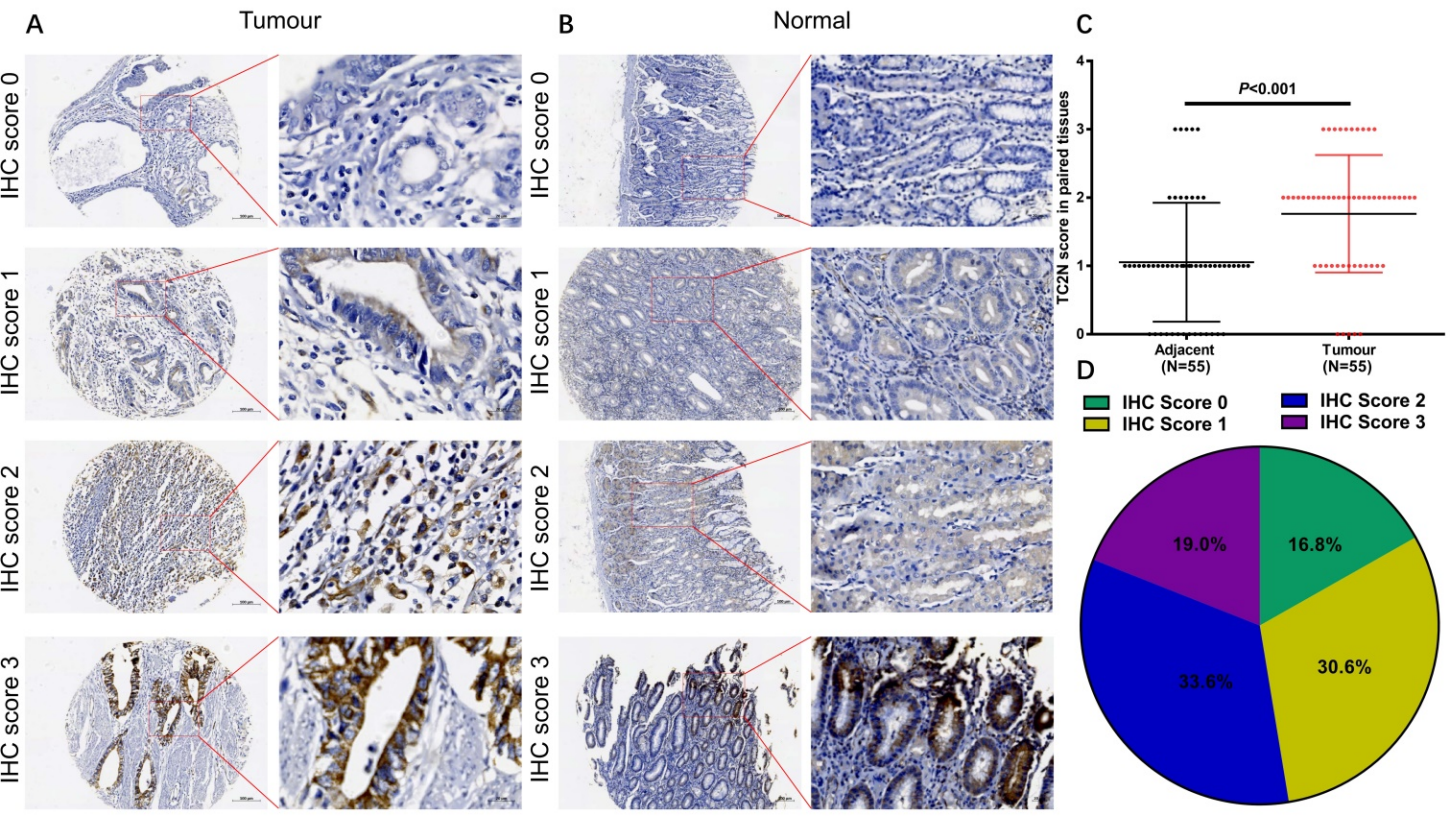
** TC2N protein expression in GC tissues and adjacent normal tissues.** (A) IHC staining of the TC2N protein in GC tissues. (B) IHC staining of the TC2N protein in adjacent normal tissues. (C) TC2N protein expression was higher in GC specimens than in adjacent normal tissues, as indicated by IHC staining. (D) Percentage of patients with GC according to TC2N protein expression as indicated by IHC scoring.

To explore the associations between TC2N expression and GC clinicopathologic characteristics, firstly, we performed IHC to detect TC2N expression in the GC tissue array, which contained 55 cases of GC tissue and paired adjacent normal tissue from GC patients. As shown in Figure 3, TC2N protein was distributed both in the nucleus and cytoplasm (Figure 3A, B). Meanwhile, we found that the protein expression of TC2N was significantly higher in primary GC compared with adjacent normal tissues (Figure 3C). Next, the expression of TC2N protein in 232 primary gastric cancer tissue samples was also detected by IHC, 55 of which was collected from the same cases with tissue array mentioned above. The patients included 159 males and 73 females, ranging in age from 27 to 83 years (mean 58.6 years). Patients were followed up for 1 to 84 months, with a median follow-up time of 51.2 months. As shown in Figure 3D, there were 71 cases (30.6%) with IHC score 1, 78 cases (33.6%) with IHC score 2, 44 cases (19.0%) with IHC score 3, and 39 cases (16.8%) with negative staining (IHC score 0). Negative and weak staining were defined as low TC2N expression (47.4%, 110/117), whereas moderate and strong staining were defined as high TC2N expression (52.6%, 122/117). The association between TC2N expression and clinicopathological features of this cohort was further evaluated. As shown in Table 1, High TC2N expression was associated with poorly differentiated histological classification (*P* < 0.001), large tumor size (*P* < 0.05), advanced distant metastasis (*P* < 0.05) and tumor-node-metastasis (TNM) stage (*P* < 0.05).

### High TC2N expression indicated worse prognosis in patients with gastric cancer

**Figure 4:**
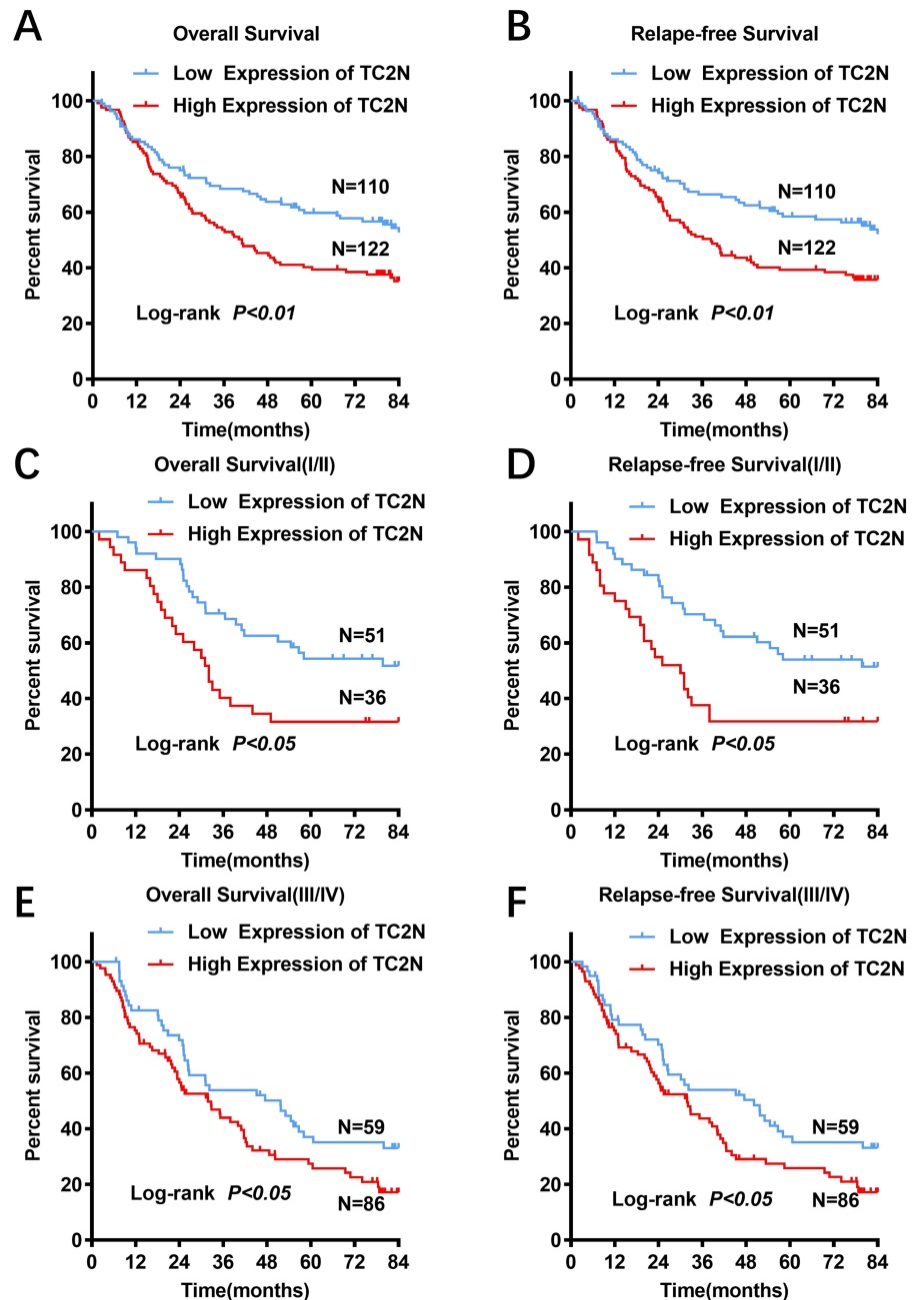
** High TC2N expression indicated worse prognosis in patients with gastric cancer.** (A-B) Patients with high expression of TC2N had a worse overall survival (A) and relapse-free survival (B) than those with low TC2N expression (*P* < 0.01). (C-D) High TC2N expression in gastric cancer tissues indicated worse overall survival (C) and relapse-free survival (D) in patients with early stage gastric cancer (TNM stage: I and II). (E-F) High TC2N expression in gastric cancer tissues predicted worse overall survival (E) and relapse-free survival (F) in patients with advanced gastric cancer (TNM stage: III and IV).

For all subjects in the present study, the follow-up period ranged from 1 to 84 months, with a median follow-up time of 51.2 months. And 123 (53.0%) patients were confirmed dead at the follow-up end point. The median overall survival time was 64.4±11.6 months and the 5-year overall survival (OS) rate was 50.7%. In GC patients with high TC2N expression, the overall 5-year survival rate was obviously poorer than that in GC patients with low expression of TC2N (39.0% vs. 59.4%; *P* < 0.001, Figure 4A). By comparing the Kaplan-Meier curves of the relapse-free survival (RFS) stratified by TC2N expression (Figure 4B), it showed the consistent results that high level of TC2N is a signal of poor prognosis in GC patients. Furthermore, we also detected the prognostic indicator effect of TC2N expression in early (TNM stage I and II) (Figure 4C, D) and advanced (TNM stage III and IV) (Figure 4E, F) gastric cancer, respectively. In early gastric cancer, the 5-year overall survival was 54.3% and 31.6% in the groups with TC2N low and high expression, respectively. And in advanced gastric cancer, the 5-year overall survival was 35.0% and 25.8% in the TC2N low and high expression groups, respectively. Similar results have been observed when RFS was used to be the comparative index. These results suggested that patients with high TC2N expression in gastric cancer tissues showed poorer prognosis than those with low TC2N expression regardless of TNM stage.

We also performed univariate Cox regression to estimate the value of prognostic evaluation of TC2N expression and the clinicopathological features for patients with gastric cancer. Univariate analysis showed that high TC2N level together with female, younger, advanced differentiation degree, large tumor size, more than two parts of stomach involved and advanced T, N, and M stage were significantly associated with poor overall survival of gastric cancer patients (Table 2). To correct the effects of confounding factors, multivariate Cox regression analysis was performed by using the method “Forward Stepwise (Conditional LR)”. It was revealed that high TC2N expression was also associated with poor survival of gastric cancer patients after adjusting for tumor size, depth of tumor invasion, lymph node metastasis and distant metastasis (HR = 1.616; 95%CI: 1.121-2.332, *p*= 0.010) (Table 2). These results indicated that the protein expression of TC2N could be an independent prognostic predictor for patients with gastric cancer.

### TC2N promotes gastric cancer cell proliferation, migration and invasion *in vitro*

**Figure 5:**
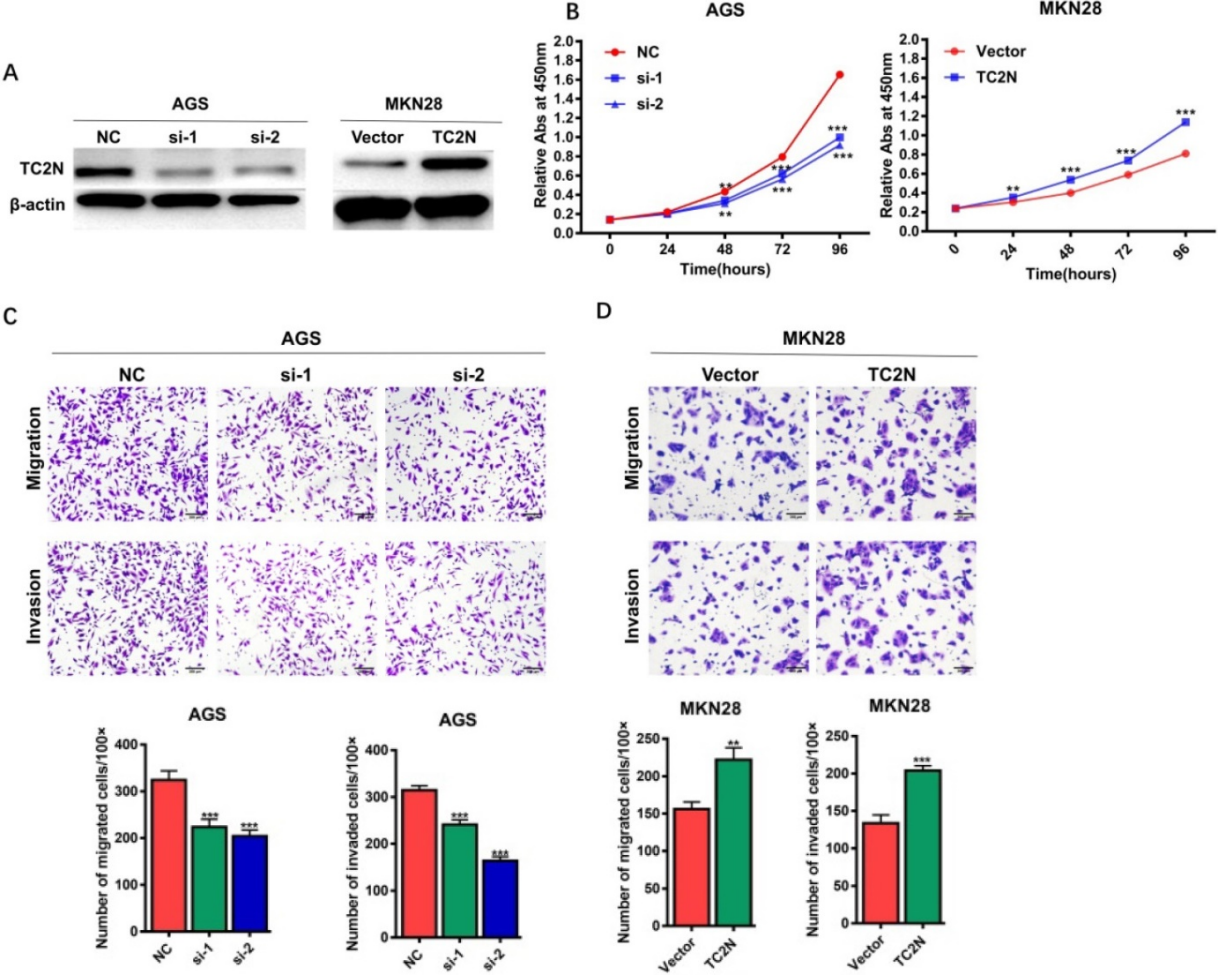
** Effects of ectopic expression of TC2N on gastric cancer cell proliferation migration and invasion *in vitro*.** (A) Knockdown of *TC2N* in AGS cells and overexpression of *TC2N* in MKN28 cells were identified by WB assay. β-actin serves as a loading control. (B) CCK8 assays were carried out in AGS cells expressing the negative control or siRNA of *TC2N* and in MKN28 cells expressing the vector control or *TC2N*. (C, D) Migration and invasion assays were carried out in AGS cells expressing the negative control or siRNA of *TC2N* and in MKN28 cells expressing the vector control or *TC2N*. **, *P* < 0.01. ***, *P* < 0.001.

To explore the potential role of TC2N in the development of GC, TC2N was knocked-down in AGS cells or overexpressed in MKN28 cells, and the expression of TC2N was verified by WB (Figure 5A). We then assessed the role of TC2N in cell proliferation by CCK8 assay (Figure 5B). The data showed that TC2N knockdown markedly impeded the proliferation of AGS cells, while TC2N overexpression promoted the growth of MKN28 cells. Next, transwell assays were performed to examine the effect of TC2N on migratory and invasive capacity of GC cells. The results showed that the downregulation of TC2N inhibited the migration and invasion of AGS cells, whereas TC2N overexpression promoted the migration and invasion of MKN28 cells (Figure 5C, D). These above results suggested that *TC2N* might act as a potential gastric cancer oncogene.

### Analysis of genes differentially expressed in correlation with *TC2N* in GC

**Figure 6:**
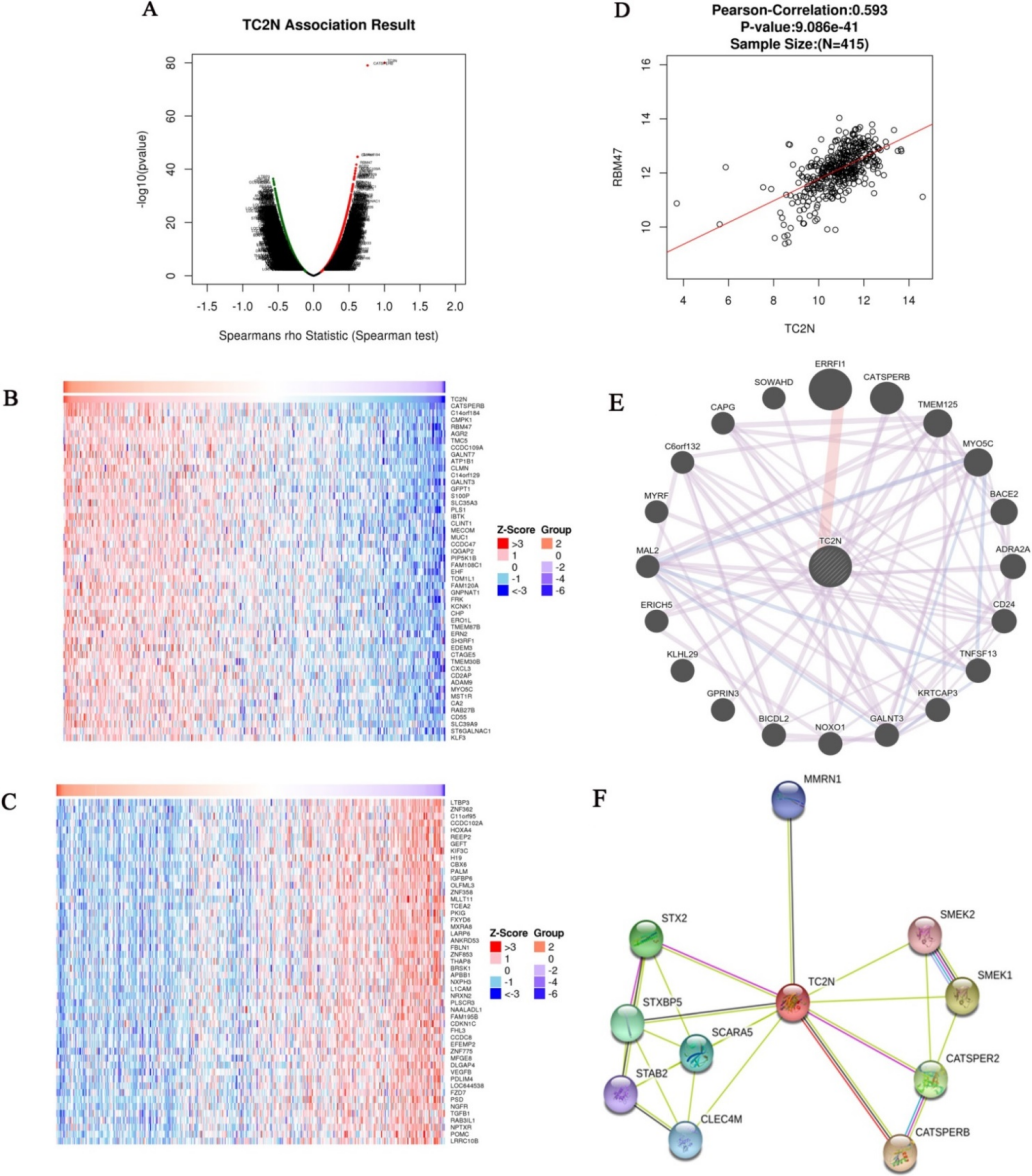
** Genes differentially expressed in correlation with *TC2N* in gastric carcinoma (LinkedOmics).** (A) A Spearman test was used to analyze correlations between *TC2N* and genes differentially expressed in GC. (B, C) Heat maps showing genes positively and negatively correlated with *TC2N* in GC (TOP 50). Red indicates positively correlated genes and green indicates negatively correlated genes. (D) The scatter plot shows Pearson correlation of *TC2N* expression with expression of *RBM47*. (E) Protein-protein interaction network of *TC2N*-target networks (GeneMANIA). (F) The interaction network of the *TC2N*-correlated genes in GC (STRING).

The Function module of LinkedOmics was used to analyze mRNA sequencing data from 415 GC patients in the TCGA. As shown in the volcano plot (Figure 6A), 3,733 genes (dark red dots) showed significant positive correlations with *TC2N*, whereas 5,420 genes (dark green dots) showed significant negative correlations (false discovery rate [FDR] < 0.01). The 50 significant gene sets positively and negatively correlated with *TC2N* as shown in the heat map (Figure 6B, C). This result suggests a widespread impact of *TC2N* on the transcriptome. We noticed that *TC2N* expression showed a strong positive association with expression of *CATSPERB* (positive rank #1, Pearson correlation = 0.75, *P* = 4.694e-76) and some other genes which have been proved to be connected with cancer, such as *GALNT3* (positive rank #2, Pearson correlation = 0.58, *P* = 5.76E-38), *RBM47* (positive rank #4, Pearson correlation = 0.60, *P* = 1.86E-42). The scatter plot shows Pearson correlation of *TC2N* expression with expression of *RBM47* (Figure 6D). In addition, we analyzed the PPI networks of TC2N by using the GeneMANIA and STRING program (Figure 6E, F), and predicted a strong interaction between the proteins of TC2N and CATSPERB, as well as the proteins of TC2N and GALNT3.

## Discussion

The present study demonstrated that high TC2N expression is evidently associated with poor prognosis and its expression may serve as a biomarker for the prognosis of patients with GC. First, we found that the expression of *TC2N* was obviously higher in GC than normal tissue by analyzing TCGA data, and we validated it in tissue specimens and cells by conducting RT-qPCR and WB. Second, we proved that TC2N protein expression was significantly higher in GC tissues than in adjacent normal tissues, as based on tissue microarrays derived from 55 patients. Meanwhile, IHC staining of tissues from 232 GC patients verified that TC2N overexpression correlated with clinicopathological features in GC and was associated with poor OS and RFS. Third, the vitro assays proved that *TC2N* promoted gastric cancer cell proliferation, migration and invasion. Finally, the analysis of genes differentially expressed in correlation with *TC2N* in GC suggested a widespread impact of *TC2N* on the transcriptome and an intensive connection between *TC2N* and genes related to human cancer and other diseases, such as* CATSPERB*, *GALNT3* and *RBM47*. Taken together, we discovered that *TC2N* may play a role of oncogene in gastric cancer.

Previous studies have reported the essential roles of protein containing the C2 domain in endocytosis, cellular metabolism and cancer 29-31. TC2N expression is also associated with the prognosis and development of human lung cancer and breast cancer 16-18. Former researchers have found that TC2N overexpression in lung cancer leads to enhancing the proliferation ability and inhibiting apoptosis through the repression of p53 function in a transcription-dependent manner. In another way, TC2N also can facilitate migration and invasion of lung cancer cells *in vitro* and promote tumor metastasis *in vivo* through increasing the degradation of IκB by promoting its phosphorylation, and subsequently activating NF-κB activity. Genes with similar expression patterns always share similar functions 32, 33. However, *TC2N* was found to play a completely different role in breast cancer. TC2N is also frequently overexpressed in breast cancer, but interestingly, high TC2N expression predicts favorable prognosis in breast cancer. Further study proved that upregulation of TC2N obviously restrained breast cancer cell proliferation *in vitro* by blocking AKT signaling in a PI3K dependent and independent way, suggesting that *TC2N* may be a tumor suppressor in breast cancer. In this study, we also demonstrated that TC2N overexpression was significantly related to the clinicopathological features in GC and promoted its development *in vitro*. Bioinformatic analysis suggested a connection between TC2N and a series of genes related to human cancer and other diseases, such as CATSPERB, a novel transmembrane protein playing role in the field of reproduction 34, GALNT3, proved to play important roles in different cancers 35-37 and RBM47, reported to inhibit tumor progression in lung, breast and colon cancer 38-40. These evidences further verified the potential role of *TC2N* in GC from another aspect. However, we did not explore the biological function of *TC2N in vivo* and the detailed mechanisms are still unclear, which is a limitation of this study.

In conclusion, our results revealed that TC2N overexpression is evidently associated with poor prognosis and promotes the development of GC. TC2N may act as an independent prognostic factor in patients with GC. Further studies are required to clarify the molecular mechanisms by which *TC2N* promotes GC development.

## Figures and Tables

**Table 1:** Relationships between TC2N expression and clinical-pathological parameters in gastric cancer

Parameters	Cases number	TC2N expression		*P* value
		Low	High	
**Gender**				0.862
Male	159 (68.5%)	76 (69.1%)	83 (68.0%)	
Female	73 (31.5%)	34 (30.9%)	39 (32.0%)	
**Age**				0.926
< 50	47 (20.3%)	22 (20.0%)	25 (20.5%)	
≥ 50	185 (79.7%)	88 (80.0%)	97 (79.5%)	
**Histological classification**				<0.001
Well and moderately differentiated	79 (34.1%)	54 (49.1%)	25 (20.5%)	
Poorly differentiated	153 (65.9%)	56 (50.9%)	97 (79.5%)	
**Tumor size**				0.014
≤ 4 cm	109 (47.0%)	61 (55.5%)	48 (39.3%)	
> 4 cm	123 (53.0%)	49 (44.5%)	74 (60.7%)	
**Tumor location**				0.944
Upper stomach	55 (23.7%)	27 (24.5%)	28 (22.9%)	
Middle stomach	34 (14.7%)	17 (15.5%)	17 (13.9%)	
Lower stomach	89 (38.4%)	40 (36.4%)	49 (40.2%)	
More than two parts	54 (23.3%)	26 (23.6%)	28 (23.0%)	
**Depth of invasion**				0.516
T1	19 (8.2%)	8 (7.2%)	11 (9.0%)	
T2	30 (12.9%)	15 (13.6%)	15 (12.2%)	
T3	137 (59.1%)	61 (55.5%)	76 (62.3%)	
T4	46 (19.8%)	26 (23.6%)	20 (16.4%)	
**Lymph node metastasis**				0.184
N0	78 (33.6%)	42 (38.1%)	36 (29.5%)	
N1	80 (34.5%)	45 (40.9%)	35 (28.7%)	
N2	41 (17.7%)	15 (13.6%)	26 (21.3%)	
N3	33 (14.2%)	8 (7.3%)	25 (20.5%)	
**Distant metastasis**				0.032
M0	182 (78.4%)	93 (84.5%)	89 (73.0%)	
M1	50 (21.6%)	17 (15.5%)	33 (27.0%)	
**TNM stage**				0.020
IA	14 (6.0%)	8 (7.3%)	6 (4.9%)	
IB	21 (9.1%)	14 (12.7%)	7 (5.7%)	
II	52 (22.4%)	29 (26.4%)	23 (18.9%)	
IIIA	47 (20.3%)	25 (22.7%)	22 (18.0%)	
IIIB	21 (9.1%)	5 (4.5%)	16 (13.1%)	
IV	77 (33.2%)	29 (26.4%)	48 (39.3%)	

^a^: The pathological diagnoses and classifications were made according to American Joint Committee on Cancer (AJCC) cancer staging manual, 7^th^ ed.

**Table 2:** Association of various factors with overall survival determined by Cox regression

Variables	Univariate Cox analysis				Multivariate Cox analysis			
	*HR*	95%*CI*		*P* value	*HR*	95%*CI*		*P* value
		Lower	Upper			Lower	Upper	
**Gender**								
Male	1							
Female	1.414	1.167	2.016	0.022				
**Age**								
< 50	1							
≥ 50	0.624	0.428	0.975	0.043				
**Histological classification**								
Well and moderately differentiated	1							
Poorly differentiated	2.016	1.679	3.183	0.001				
**Tumor size**								
≤ 4 cm	1				1			
> 4 cm	2.138	1.152	4.126	<0.001	1.573	1.315	2.422	0.029
**Tumor location**				0.007				
Upper stomach	1							
Middle stomach	0.945	0.527	1.893	0.981				
Lower stomach	0.695	0.621	1.190	0.238				
More than two parts	1.239	1.311	2.678	0.039				
**Depth of invasion^a^**				<0.001				0.006
T1	1				1			
T2	1.489	0.318	8.812	0.622	2.012	0.413	9.879	0.398
T3	8.786	2.056	28.745	0.003	5.622	1.445	22.423	0.018
T4	22.773	5.023	92.101	<0.001	7.691	1.698	36.012	0.007
**Lymph node metastasis^a^**				<0.001				0.003
N0	1				1			
N1	2.123	1.129	3.147	0.004	1.891	0.801	2.234	0.901
N2	6.012	3.761	9.012	<0.001	2.712	1.654	5.812	0.001
N3	7.071	3.613	10.972	<0.001	2.231	1.414	3.914	0.012
**Distant metastasis^a^**								
M0	1				1			
M1	3.418	3.011	6.746	<0.001	2.087	1.294	3.294	0.003
**TC2N level**								
Low	1				1			
High	2.003	1.354	2.691	<0.001	1.451	1.312	2.241	0.013

^a^: The pathological diagnoses and classifications were made according to American Joint Committee on Cancer (AJCC) cancer staging manual, 7^th^ ed.

## Abbreviations

*TC2N*: Tac2-N
GC: Gastric cancer
TCGA: The Cancer Genome Atlas
GEPIA: Gene Expression Profiling Interactive Analysis tools
RT-qPCR: RNA isolation and reverse transcription-quantitative real-time PCR
WB: Western blot
IHC: Immunohistochemical
PPI: Protein-protein interaction
OS: Overall survival
RFS: relapse-free survival
