# Overexpression of TC2N is associated with poor prognosis in gastric cancer: Erratum

**DOI:** 10.7150/jca.73345

**Published:** 2022-04-25

**Authors:** Jianbo Xu, Xinde Ou, Jin li, Qinbo Cai, Kaiyu Sun, Jingning Ye, Jianjun Peng

**Affiliations:** 1 Department of Gastrointestinal Surgery, the First Affiliated Hospital, Sun Yat-sen University, 58 Zhongshan 2nd Road, Guangzhou 510080, China.; 2 Digestive Disease Center, the Seventh Affiliated Hospital, Sun Yat-sen University, 628 Zhenyuan Road, Shenzhen 518000, China.; 3 Laboratory of General Surgery, the First Affiliated Hospital, Sun Yat-sen University, 58 Zhongshan 2nd Road, Guangzhou 510080, China.

In our paper 1, the western blot band for TC2N in paired gastric cancer tissues and matched normal adjacent mucosa was used wrong in Figure 2E, so we replaced with the correct band. We are deeply sorry and sincerely apologize for the error and for any inconvenience that may cause to the readers and the editors of this journal. Figure 2E was corrected as follows.

## Figures and Tables

**Figure 2:**
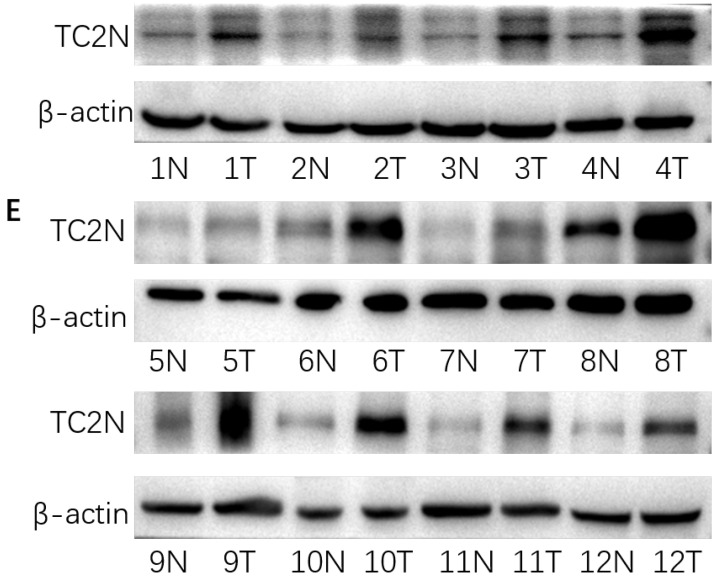
High TC2N expression in gastric cancer. (A) Analysis of TC2N expression in unpaired GC (T = 408) and normal tissues (N = 211) in the GEPIA (P<0.05). (B, C) The measurements of TC2N expression in gastric cancer cell lines compared with the normal cell line GES1 by qRT-PCR(B) and WB(C). (D, E) The measurements of TC2N expression in 12 paired GC tissues and matched normal adjacent mucosa, analyzed by qRT-PCR(D) and WB(E). T, GC tissues; N, matched normal adjacent mucosa. *P < 0.05.

## References

[1] Xu J, Ou X, Li J, Cai Q, Sun K, Ye J, Peng J (2021). Overexpression of TC2N is associated with poor prognosis in gastric cancer. J Cancer.

